# Analgesic effect of ultrasound-guided erector spinae plane block (espb) in general anesthesia for cesarean section: a randomized controlled trial

**DOI:** 10.1186/s12871-022-01781-8

**Published:** 2022-08-02

**Authors:** Jia hu, Qi Chen, Qian Xu, Yun Song, Ke Wei, Xiao-feng Lei

**Affiliations:** 1 Department of Anesthesiology, Woman and Children’s Hospital of Chongqing Medical University, Chongqing, China; 2 Department of Anesthesiology, Chongqing Health Center for Woman and Children, 120# Longshan Road， Yubei District, Chongqing, China; 3 Department of Anesthesiology, The First Affiliated Hospital of Chongqing Medical University, Chongqing, China; 4 Department of Anesthesiology, Chongqing University Cancer Hospital, Chongqing, China

**Keywords:** Erector spinae plane block, Ultrasound-guided, General anesthesia, Cesarean section

## Abstract

**Background:**

The analgesic effects of erector spinae plane block in general anesthesia for cesarean section and recovery from puerperae remain unclear.

**Methods:**

Sixty patients with contraindications for spinal anesthesia who required general anesthesia for cesarean section were enrolled and randomly divided into the erector spinal plane block (ESPB) combined with the general anesthesia group (group E) and general anesthesia group (group G). Group E received bilateral ESPB (20 ml of 0.25% ropivacaine on each side) under ultrasound guidance 30 min before general anesthesia. The primary outcomes were the number of patient-controlled intravenous analgesia (PCIA) boluses, and Bruggemann comfort scale (BCS) scores at 2 h, 6 h, 12 h, and 24 h after operation. The second outcome was intraoperative anesthesia dosage, fetal delivery time, puerperae emergence time, visual analog scale (VAS) at 2 h, 6 h, 12 h, and 24 h after operation, and incidence of nausea and vomiting. Heart rate (HR) and mean arterial pressure (MAP) were recorded 10 min before the start of anesthesia (T0), at the induction of anesthesia (T1), at skin incision (T2), and fetal delivery (T3), and immediately after surgery (T4).

**Results:**

The number of PCIA boluses was lower in group E than in group G (*P* < 0.001). The BCS score increased at 2 h and 6 h after the operation in group E (*P* < 0.05), while the VAS score significantly decreased in group E at the same time (*P* < 0.05). Compared with group G, the doses of propofol and remifentanil were significantly decreased in group E (*P* < 0.001), the emergence time of puerperae was shortened (*P* = 0.003), and the incidence of nausea and vomiting was significantly decreased (*P* = 0.014).

**Conclusion:**

Ultrasound-guided ESPB applied to general anesthesia for a cesarean section can significantly reduce the required dose of general anesthetic drugs, shorten the recovery time of the puerperae, and improve postoperative analgesia.

**Trial registration:** www.clinicaltrials.gov under the number ChiCTR2200056337 (04–02-2022).

## Background

Several studies have shown China’s cesarean section rate has been high in recent years [1]. Spinal anesthesia is still the preferred anesthesia method for cesarean section, with perfect analgesia, good muscle relaxation, and no adverse effects on the mother or fetus [2, 3]. General anesthesia is suitable for puerperae that are prohibited from spinal anesthesia, especially for those with coagulation disorders, thrombocytopenia, spinal deformity, and other complications. However, it is necessary to maintain a good depth of anesthesia and sufficient analgesia and to consider the impact of anesthesia drugs on the fetus [4]. Total intravenous anesthesia is widely used; however, multimodal analgesia is required to reduce drug use and side effects [5]. Nerve blocks currently used for multimodal analgesia include ultrasound-guided transversus abdominis plane, quadratus lumborum plane, and erector spinae plane blocks [6, 7].

The erector spinal plane block (ESPB) was initially described by Forero et al. to achieve thoracic analgesia during the T5 transverse process [8]. The block is achieved by injecting an anesthetic into the plane between the erector spinae muscle and the transverse process, which can be achieved by blocking the dorsal and lateral branches of the spinal nerve. Recently, ESPB has been widely used in thoracic and lumbar spinal surgery [9, 10]. Few studies have investigated the analgesic effects of ESPB in obstetrics.

This study aimed to evaluate the clinical effect of ESPB in general anesthesia for cesarean section and to investigate whether it would be a part of a multimodal opioid-sparing analgesia procedure in cesarean section.

## Methods

### Patient enrollment

**Fig 1:**
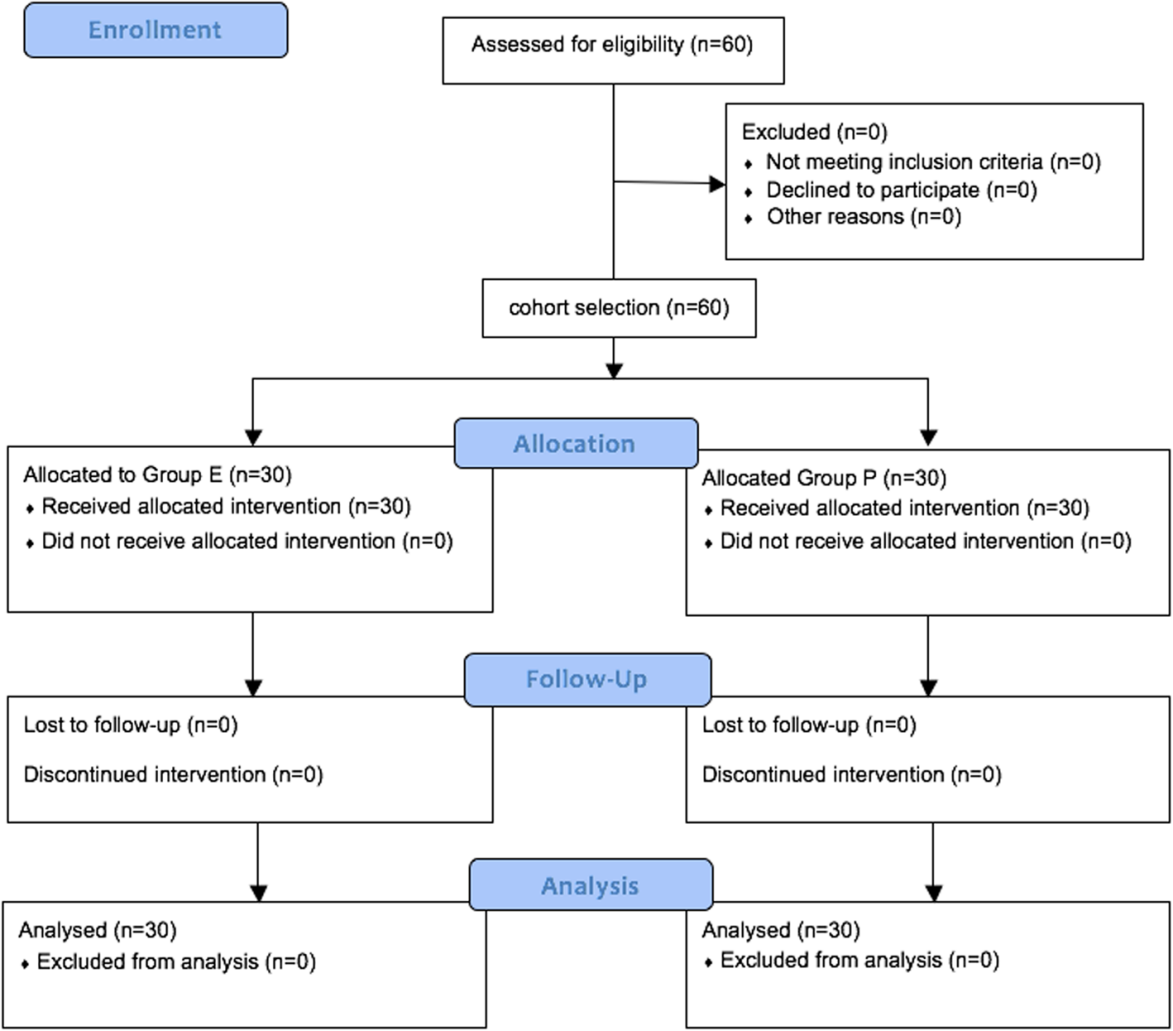
CONSORT flow diagram showing the number of patients at each phase of the study

Sixty participants in our hospital were contraindicated for spinal anesthesia and required general anesthesia for a cesarean section from March 1, 2022, to April 10, 2022, and were selected. The participants were randomly divided into two groups according to a 1:1 ratio through the central randomization network system: the ESPB combined with the general anesthesia group (Group E) and the general anesthesia group (Group G) (Fig. 1). This study was approved by the Ethics Committee of Women and Children's Hospital of Chongqing Medical University and registered at www.clinicaltrials.gov under the number ChiCTR2200056337(04–02-2022), following the tenets of the Declaration of Helsinki. Written informed consent was obtained from all the participants.

The inclusion criteria were as follows: (1) singleton pregnancy for elective cesarean section; (2) spinal anesthesia prohibited (due to reasons such as spinal deformity, spinal trauma, and coagulation dysfunction); (3) American Society of Anesthesiologists grade II; and (4) normal cognitive function.

The exclusion criteria were as follows: (1) allergy to local anesthetics; (2) body mass index (BMI) > 35 kg/m^2^; and (3) severe heart disease, hypertension, diabetes, etc.

### Patient Management

General anesthesia in both groups was performed by total intravenous anesthesia, and anesthesia induction was performed with 4–6 μg/ml propofol and 4–6 ng/ml remifentanil TCI target-controlled infusion, 0.6 mg/kg rocuronium before endotracheal intubation. Anesthesia was maintained with 2.5–4 µg/ml propofol and 4–6 ng/ml remifentanil TCI target-controlled infusion. The utility of BIS for sedation management during monitored anesthesia care was maintained within the range of 40–60. After the fetus was delivered, sufentanil 0.2 μg/kg was injected intravenously, and rocuronium was administered during the operation as needed. All patients were transferred to the post-anesthesia care unit after surgery. The patient-controlled intravenous analgesia (PCIA) formula comprised fentanyl 0.2 mg + tramadol 80 mg + dexamethasone 10 mg to 100 mL 0.9% normal saline, with a 2 mL/h background infusion and a 2 mL bolus dose, with a lock time of 15 min.

Group E: Thirty minutes before induction of general anesthesia, bilateral ESPB was performed under ultrasound guidance by the same anesthesiologist in the preparation room for anesthesia. The patients were instructed to remain in the lateral decubitus position. The ultrasound probe was placed on the spinous process of the 9th thoracic vertebra along the short axis. Subsequently, the probe was slid slightly in the lateral direction until the transverse processed image was visible. With subcutaneous infiltration of 3 mL of 2% lidocaine, a 22G blunt needle (Spinocan, B. Braun Melsungen AG, Germany) was introduced from the outside toward the transverse process (T9) using the in-plane method until the needle tip crossed all the muscles. Subsequently, 20 ml of 0.25% ropivacaine was injected between the transverse process and the deep surface of the erector spinae on each side.

Group G: with no ESPB.

### Pain measurements and outcomes

Heart rate (HR) and mean arterial pressure (MAP) were recorded 10 min before the start of anesthesia (T0), at the induction of anesthesia (T1), at skin incision (T2), at fetal delivery (T3), and at the end of the operation (T4). The intraoperative propofol and remifentanil dosages, fetal delivery time, and emergence time were recorded. The VAS and BCS comfort scores of patients were measured at 2 h, 6 h, 12 h, and 24 h after the operation. The VAS score is ranked on a point system from 0–10; 0 points: no pain; less than 3 points: mild pain, which the patient can tolerate; 4–6 points: pain that affects sleep but can be tolerated; 7–10 points: the patient has increasingly severe pain that is unbearable. The BCS comfort score was ranked as follows: 0, persistent pain; 1, no pain at rest; severe pain when breathing or coughing; 2, no pain at rest; mild pain during breathing or coughing; 3, no pain during deep breathing; 4, no pain during deep breathing and coughing; record the number of PCA boluses within 24 h after surgery; and record the incidence of nausea and vomiting within 24 h after the operation. All data were obtained by the same anesthesiologist, who was blinded to the group assignment and was not involved in implementing the nerve block.

### Statistical analysis

The sample size estimation was based on the number of PCA boluses within 24 h of the pilot study by our team. To detect a 20% change in PCA boluses with an error of 0.05 and a power of 80%, the minimum sample size was found to be 23 patients per group. Thirty patients were enrolled in each group to account for dropouts.

SPSS 20.0 statistical software was used for data analysis, normally distributed measurement data were expressed as mean ± standard deviation (intraoperative propofol and remifentanil dosages, fetal delivery time, and emergence time) or as number (%) (incidence of nausea and vomiting), paired t-test was used for intra-group comparison, group t-test was used for inter-group comparison. A two-way repeated- measures analysis of variance with the Bonferroni post hoc -test was used to compare the VAS, hemodynamic variables, and BCS within and between the two groups. Statistical significance was defined as *P* < 0.05.

## Results

**Fig 2:**
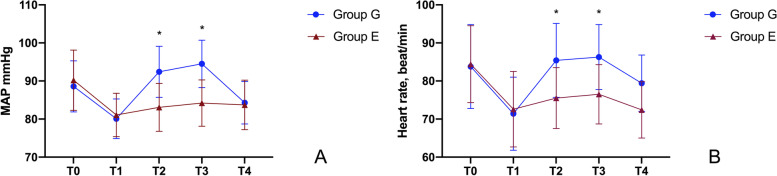
Changes in MAP and HR between the two groups

There was no significant difference in age, weight, height, or gestational age between the two groups of patients, as shown in Table 1. Compared with T1, T2, and T3 in group G, MAP and HR were significantly increased (*P* < 0.05) and were higher than those in group E at the same time point (*P* < 0.05), and there was no statistically significant difference between the T1, T2, and T3 groups in group E (*P* > 0.05) (Fig. 2).

**Table 1:** Characteristics

Variables	Group G(***n*** = 30)	Group E(***n*** = 30)	*P*
Age, yr	28.4 ± 4.7	28.8 ± 4.6	0.336
Height, cm	160.7 ± 4.5	159.4 ± 4.0	0.562
Weight, kg	69.1 ± 9.8	68.3 ± 9.1	0.295
Gestational weeks	38.8 ± 1.3	38.7 ± 1.2	0.671

The dosages of propofol and remifentanil in group E were significantly lower than those in group G (*P* < 0.001), and the emergence of group E was significantly shorter than that in group G (*P* = 0.003). There was no significant difference in the fetal delivery time between the two groups (*P* > 0.05) (Table 2).

**Table 2:** Intraoperative and postoperative related indicators

Variables	Group G(***n***=30)	Group E(***n***=30)	***P***
propofol, mg	478.8±69.0	413.2±52.9^a^	<0.001
remifentanil, ug	523.7±69.0	446.9±57.1^a^	<0.001
delivery time, min	5.0±1.5	4.8±1.7	0.307
emergence time, min	12.1±4.6	9.8±3.5">^a^	0.003
bolus of PCIA	11.2±2.1	5.3±1.7	<0.001
PONV	7(23.3%)	3(10%)	0.014

^a^statistical difference when compared with group G

Table 2 also compares PCA use and the incidence of nausea and vomiting between the two groups. Compared to group G, group E required fewer bolus doses, and the incidence of nausea and vomiting significantly decreased (*P* < 0.014).

**Fig 3:**
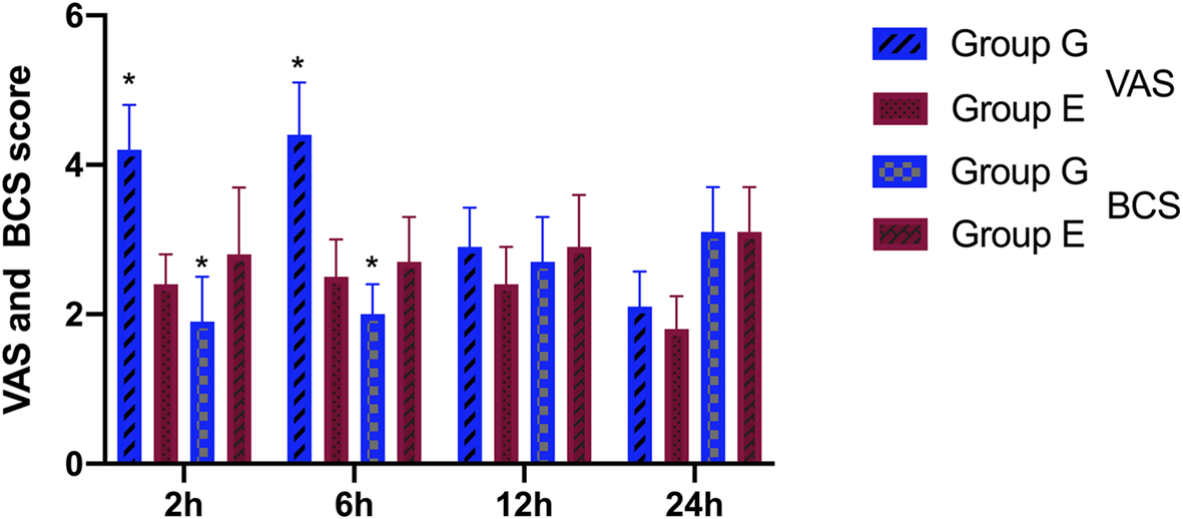
Changes in VAS and BCS between the two groups

The comparison of the VAS and BCS comfort scores between the two groups is shown in Fig. 3. Compared with group G, the VAS score of group E was significantly lower at 2 h and 6 h after the operation, while the BCS comfort score was significantly higher (*P* < 0.05).

## Discussion

This study evaluated the analgesic effect of ESPB in general anesthesia for cesarean section as part of a multimodal opioid-sparing analgesia procedure. We found that erector spinae plane block combined with general anesthesia could reduce the use of intraoperative and postoperative analgesics and improve postoperative comfort compared with general anesthesia alone. Meanwhile, multimodal analgesia with the addition of erector spinae plane blocks could also obtain more stable intraoperative hemodynamic indicators and reduce the incidence of postoperative nausea and vomiting.

For women who have contraindications to spinal anesthesia and can only choose general anesthesia, the choice of anesthetic drugs and postoperative analgesia is particularly important. It is necessary to meet the appropriate depth of anesthesia, suppress the stress response of surgery, and avoid maternal awareness during surgery. Considering the potential dangers of general anesthetic drugs to mothers and babies, the safety of neonates in general anesthesia obstetrics has always been a research hotspot [11]. Anesthetics easily pass through the placenta, and higher doses of general anesthetics may inhibit neonatal breathing [12]. Many studies have been conducted to determine whether general anesthesia affects neonatal neurobehavioral abilities; however, the results are conflicted. Therefore, the anesthetic dosage should be minimized to avoid fetal damage. Local anesthesia is an important component of multimodal analgesia, with limited effects, precise effects, and little impact on the mother and baby [13]. In addition, studies have shown that the neurotoxicity of ropivacaine in TAPB during cesarean section may be related to increased plasma protein binding of ropivacaine during pregnancy [14]. Therefore, in this study, ESPB was selected for the ninth thoracic vertebrae, and 20 ml of ropivacaine at a low concentration of 0.25% was unilaterally administered to ensure the block effect and prevent neurotoxicity.

Currently, the erector spinae plane block is widely used in clinical practice, whether for perioperative analgesia or chronic pain management. Frassanito et al. used ESPB in laparoscopic hysterectomy, which can reduce the dosage of anesthetics, shorten recovery time, and relieve postoperative pain [15]. Postmastectomy pain syndrome (PMPS) is a common complication that occurs in 20%-44% of patients after breast surgery and is often challenging to manage. Hasoon et al. verified that ESPB could be utilized to provide analgesia to patients suffering from this difficulty in managing the condition [16]. Meanwhile, some studies compared ESPB with other nerve blocks in cesarean section, but the results differed. Hamed et al. confirmed that ESPB has a successful postoperative analgesic effect and may limit opioid consumption in parturients compared to intrathecal morphine (ITM) for analgesia after elective cesarean delivery under spinal anesthesia [17]. Boules and Malawat verified that the ESP block provided prolonged analgesia with a significant decrease in analgesic requirement compared with the TAP block [18, 19]. However, a recent Bayesian network meta-analysis showed that erector spinae block could reduce pain scores at 6 and 12 h. Still, TAPB is the most comprehensive local anesthetic technique for postoperative cesarean section analgesia [5].

Our research has some limitations. The sample size was small, and we strive to conduct a larger sample study and a deeper discussion on the mechanism of action in the future.

In conclusion, ESPB used in general anesthesia for cesarean section can significantly reduce the dosage of general anesthesia, shorten the recovery time, improve the postoperative analgesic effect, and improve the perioperative comfort of the puerpera while ensuring the safety of mothers and their babies.

## Data Availability

The data analyzed and preserved during the current study are available from the corresponding author upon reasonable request via e-mail.

## Abbreviations

ESPB: Erector spinae plane block
HR: Heart rate
MAP: Mean arterial pressure
VAS: Visual analog scale
BCS: Bruggemann comfort scale
PCIA: Patient-controlled intravenous analgesia;
ESM: Erector spinae muscle
PCIA: Patient-controlled intravenous analgesia
PONV: Postoperative nausea and vomiting

## References

[1] Li L, Cui H (2021). The risk factors and care measures of surgical site infection after cesarean section in China: A retrospective analysis. BMC Surg.

[2] Ngan WD, Lee SW, Ng FF, Tan PE, Khaw KS (2015). Randomized double blinded comparison of norepinephrine for maintenance of blood pressure during spinal anesthesia for cesarean deliverys. Anesthesiology.

[3] Juang J, Gabriel RA, Dutton RP, Palanisamy A, Urman RD (2017). Choice of anesthesia for Cesarean delivery: An analysis of the national anesthesia clinical outcomes registry. Anesth Analg.

[4] Cobb BT, Lane-Fall MB, Month RC, Onuoha OC, Srinivas SK, Neuman MD (2019). Anesthesiologist specialization and use of General Anesthesia for Cesarean delivery. Anesthesiology.

[5] Wang J, Zhao G, Song G, Liu J (2021). The efficacy and safety of local anesthetic techniques for postoperative analgesia After Cesarean section: A Bayesian network meta-analysis of randomized controlled trials. J Pain Res.

[6] Roofthooft E, Joshi GP, Rawal N, Van de Velde M, PROSPECT Working Group* of the European Society of Regional Anaesthesia and Pain Therapy and supported by the Obstetric Anaesthetists’ Association (2021). PROSPECT guideline for elective caesarean section: Updated systematic review and procedure-specific postoperative pain management recommendations. Anaesthesia..

[7] Hussain N, Brull R, Weaver T, Zhou M, Essandoh M, Abdallah FW (2021). Postoperative analgesic effectiveness of quadratus lumborum block for Cesarean delivery under spinal anesthesia. Anesthesiology..

[8] Forero M, Adhikary SD, Loppe ZH, Tsui C, Chin KJ (2016). The erector spinae plane block: A novel analgesic technique in thoracic neuropathic pain. Reg Anesth Pain Med.

[9] Qiu Y, Zhang TJ, Hua Z (2020). Erector spinae plane block for lumbar spinal surgery: A systematic review. J Pain Res.

[10] Finnerty DT, McMahon A, McNamara JR, Hartigan SD, Griffin M, Buggy DJ (2020). Comparing erector spinae plane block with serratus anterior plane block for minimally invasive thoracic surgery: A randomised clinical trial. Br J Anaesth.

[11] Mishra PK, Yadav JBS, Singh AK, Singh RB (2020). Comparison of intravenous nalbuphine and paracetamol on Maternal hemodynamic Status, Neonatal Apgar score, and postoperative Pain given before Induction of General Anesthesia for Elective Cesarean Section. Anesth Essays Res.

[12] Tumukunde J, Lomangisi DD, Davidson O, Kintu A, Joseph E, Kwizera A (2015). Effects of propofol versus thiopental on Apgar scores in newborns and perioperative outcomes of women undergoing emergency cesarean section: A randomized clinical trial. BMC Anesthesiol.

[13] El-Boghdadly K, Desai N, Halpern S, Blake L, Odor PM, Bampoe S (2021). Quadratus lumborum block vs. transversus abdominis plane block for caesarean delivery: A systematic review and network meta-analysis. Anaesthesia.

[14] Forero M, Rajarathinam M, Adhikary S, Chin KJ (2017). Continuous erector spinae plane block for rescue analgesia in thoracotomy after epidural failure: A case report. A A Case Rep.

[15] Frassanito L, Zanfini BA, Catarci S, Sonnino C, Giuri PP, Draisci G (2020). Erector spinae plane block for postoperative analgesia after total laparoscopic hysterectomy: Case series and review of the literature. Eur Rev Med Pharmacol Sci.

[16] Hasoon J, Urits I, Viswanath O, Dar B, Kaye AD (2021). Erector spinae plane block for the treatment of post mastectomy pain syndrome. Cureus.

[17] Hamed MA, Yassin HM, Botros JM, Abdelhady MA (2020). Analgesic efficacy of erector spinae plane block compared with intrathecal morphine After elective Cesarean section: A prospective randomized controlled study. J Pain Res.

[18] Boules ML, Goda AS, Abdelhady MA, Abu El-NourAbd El-Azeem SA, Hamed MA (2020). Comparison of analgesic effect Between erector spinae plane block and transversus abdominis plane block After elective Cesarean section: A prospective randomized single-blind controlled study. J Pain Res.

[19] Malawat A, Verma K, Jethava D, Jethava DD (2020). Erector spinae plane block and transversus abdominis plane block for postoperative analgesia in cesarean section: A prospective randomized comparative study. J Anaesthesiol Clin Pharmacol.

